# Identification of Dihydrolipoamide Dehydrogenase as Potential Target of Vemurafenib-Resistant Melanoma Cells

**DOI:** 10.3390/molecules27227800

**Published:** 2022-11-12

**Authors:** Claudio Tabolacci, Deborah Giordano, Stefania Rossi, Martina Cordella, Daniela D’Arcangelo, Federica Moschella, Stefania D’Atri, Mauro Biffoni, Angelo Facchiano, Francesco Facchiano

**Affiliations:** 1 Department of Oncology and Molecular Medicine, Istituto Superiore di Sanità, Viale Regina Elena 299, 00161 Rome, Italy; 2 National Research Council, Institute of Food Science, Via Roma 64, 83110 Avellino, Italy; 3 Laboratory of Molecular Oncology, IDI-IRCCS, 00167 Rome, Italy

**Keywords:** melanoma, BRAFi resistance, targeted therapy, proteomics, protein structure, dihydrolipoamide dehydrogenase, CPI-613

## Abstract

Background: Despite recent improvements in therapy, the five-year survival rate for patients with advanced melanoma is poor, mainly due to the development of drug resistance. The aim of the present study was to investigate the mechanisms underlying this phenomenon, applying proteomics and structural approaches to models of melanoma cells. Methods: Sublines from two human (A375 and SK-MEL-28) cells with acquired vemurafenib resistance were established, and their proteomic profiles when exposed to denaturation were identified through LC-MS/MS analysis. The pathways derived from bioinformatics analyses were validated by in silico and functional studies. Results: The proteomic profiles of resistant melanoma cells were compared to parental counterparts by taking into account protein folding/unfolding behaviors. Several proteins were found to be involved, with dihydrolipoamide dehydrogenase (DLD) being the only one similarly affected by denaturation in all resistant cell sublines compared to parental ones. DLD expression was observed to be increased in resistant cells by Western blot analysis. Protein modeling analyses of DLD’s catalytic site coupled to in vitro assays with CPI-613, a specific DLD inhibitor, highlighted the role of DLD enzymatic functions in the molecular mechanisms of BRAFi resistance. Conclusions: Our proteomic and structural investigations on resistant sublines indicate that DLD may represent a novel and potent target for overcoming vemurafenib resistance in melanoma cells.

## 1. Introduction

Cutaneous melanoma, a melanocyte-originated skin cancer, ranks among the most aggressive human cancers, and its worldwide incidence has been steadily increasing over the last two decades [1,2]. Patients with early-stage melanoma can be successfully treated with surgical resection of the tumor; however, once cutaneous melanoma has metastasized, primary and acquired drug resistance strongly limits the efficacy of systemic therapies. Therefore, the development of advanced molecularly targeted therapies and new treatment approaches, including multidrug regimens, are of great clinical relevance [3,4]. Nowadays, the use of immunotherapeutic drugs like nivolumab and pembrolizumab (anti-PD-1 monoclonal antibodies), and ipilimumab (anti-CTLA-4 monoclonal antibody), as well as therapies specifically targeting oncogenic driver mutations, have revolutionized the management of malignant melanoma [5,6], but there is still a pressing need to improve the available treatments. Oncogenic mutations in BRAF, a serine/threonine–protein kinase that regulates cell proliferation and survival via the RAS/RAF/MAPK signaling pathway, occur in approximately 50% of cutaneous melanoma [7], and are also involved in other types of cancer [8]. The most frequent BRAF mutation (>90% of BRAF-mutated tumors) consists of the substitution of a valine with a glutamic acid in the kinase activation domain, at the amino acidic residue 600 (V600E) [9]. Although selective BRAF inhibitors (BRAFi), approved for clinical use, significantly improve the response rate and survival of melanoma patients, disease progression within 6–8 months from the start of therapy occurs in most cases [10]. Furthermore, despite the use of BRAFi + MEKi combinations increasing the response rate and survival outcomes, drug resistance remains the major factor limiting the clinical efficacy of targeted therapies [11,12].

Multiple mechanisms involved in BRAFi resistance have been identified, including genetic and epigenetic ones [13]. Treatment with BRAFi is associated with secretion of soluble molecules (cytokines/chemokines and growth factors) by tumor and/or tumor microenvironment (TME) cells whose autocrine and paracrine effects lead to microenvironment modifications and tumor immunogenicity alterations [14,15,16]. This interplay between immune and melanoma cells can also influence metabolic pathways, altering immune response [17]. There is a body of data supporting the notion that TME alterations and therapeutic interventions are able to modify the energetic metabolism of melanoma cells [18]. It is interesting to note that mitochondrial biogenesis, coupled with stimulation of oxidative phosphorylation, occurs in intrinsically resistant BRAF^V600E^ melanoma in order to respond to MAPK inhibitors [19]. Moreover, the metabolic plasticity of melanoma cells, in particular the shift from glycolysis to a mitochondrial respiration, also represents a well-accepted mechanism in the acquisition of resistance to BRAFi [20,21]. Therefore, the increased dependence of resistant cells to mitochondrial metabolism may represent a possible target for new therapeutic interventions. It is noteworthy that, although genomic profiling of cutaneous melanoma represents an important tool for improving patients’ stratification and management and promoting the development of targeted therapies [22], proteomic analysis makes it possible to better investigate adaptive and functional mechanisms related to BRAFi resistance [23,24]. Moreover, there are many lines of evidence underscoring that post-translational modifications (PTMs) of proteins play a crucial role in drug resistance, interfering with drug target modifications, epithelial-mesenchymal transition, and metastasis [25,26].

In the present study, we applied a mass spectrometry-based proteomic approach coupled with a multi-denaturation protocol on two different human melanoma cell lines resistant to the BRAFi vemurafenib, along with their drug-sensitive counterparts, in order to reveal structural alterations in cellular components (due, for instance, to post-translational processes) potentially related to drug resistance. The two cell lines were selected as being representative of two different phenotypes of aggressiveness (see the Materials and Methods section). Specifically, a differential denaturation protocol (TRIDENT) was applied to both the parental and resistant melanoma cell extracts in order to achieve their proteomic profiles and to also characterize them from a structural point of view [27]. These proteomic profiles were then analyzed and compared on the basis of Gene Ontology (GO) and functional pathway bioinformatics studies. This proteomic and bioinformatic approach, aimed at finding a consensus between two resistant cell lines and their own parental counterparts, suggested to us that dihydrolipoamide dehydrogenase (DLD or DLDH; EC 1.8.1.4), the subunit involved in the assembly and function of many enzymatic complexes with crucial functions in cell metabolic processes [28], could represent a novel therapeutic target. In fact, DLD is the E3 component of the three so-called mitochondrial α-ketoacid dehydrogenase complexes: the 2-oxoglutarate (α-ketoglutarate) dehydrogenase complex (OGDC), the branched-chain 2-oxoacid dehydrogenase complex (BCKDC), and the pyruvate dehydrogenase complex (PDC) [28]. Specifically, we demonstrated that DLD plays a key role in the proliferation of vemurafenib-resistant cells on the basis of structural analyses and the use of in silico tools for protein modeling.

## 2. Results

### 2.1. Comparative Evaluation of Proteomes Expressed by Vemurafenib-Sensitive and -Resistant Cells

**Figure 1:**
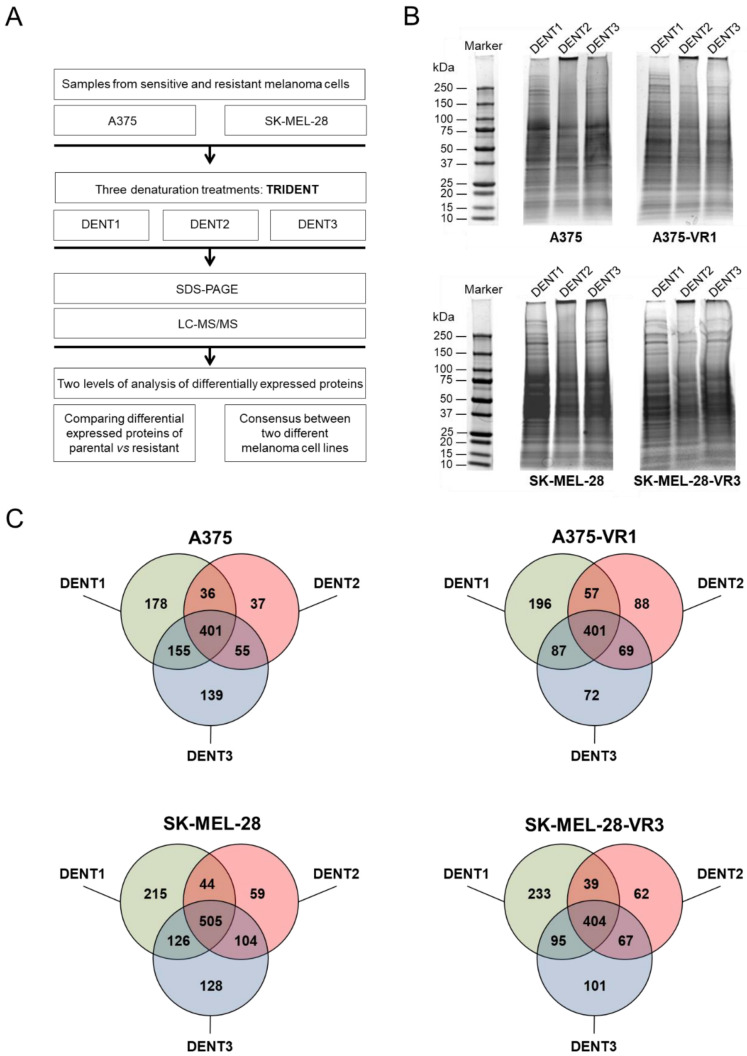
Comparative proteomic analysis of melanoma cells resistant and sensitive to vemurafenib. (**A**) Schematic representation of the experimental and analytical workflow for this study. (**B**) Representative electrophoretic separation of cell lysates after application of TRIDENT differential denaturation protocol. Samples were run on pre-cast gradient gel and stained by Coomassie Blue G-250. Different protein bands were detected after DENT1, DENT2, and DENT3 pre-treatments. (**C**) The entire protein gel lanes were excised, cut, and trypsin-digested, and proteins were identified through LC-MS/MS analysis (LTQ). Venn diagrams show the numbers of proteins overlapping among the three different denaturation protocols for A375 and SK-MEL-28 parental and resistant cells.

For the present study, resistant cell lines were generated by chronic exposure of SK-MEL-28 and A375 cells to vemurafenib. Two independent sublines (namely VR2 and VR3) for SK-MEL-28 were generated and validated as previously described [16]. The acquired resistance of A375 sublines (namely VR1 and VR2) was confirmed by SRB assay (Figure S1). To identify any relevant difference between parental cell lines and their first-established resistant sublines (SK-MEL-28-VR3 and A375-VR1) related to BRAFi resistance, the TRIDENT proteomic approach, developed to improve the analysis of complex mixtures of proteins like those in serum [27], was employed to analyze cell proteomes (Figure 1A). As illustrated in Figure 1B, in which the three different lanes represent three different pre-treatments, marked differences in electrophoretic profiles were detected when comparing the same cell lysate exposed to the different denaturation protocols, as well as when comparing lysates from parental and resistant cells. Accordingly, protein identification by mass spectrometry analysis (Figure 1C, and Tables S1–S4) revealed marked differences in protein expression patterns between drug-resistant sublines and their parental counterparts. This demonstrates that such denaturation protocols were able to explore the structure and accessibility of the cell proteomes under investigation in depth.

### 2.2. Bioinformatic Analysis of Total Differentially Expressed Proteins

**Figure 2:**
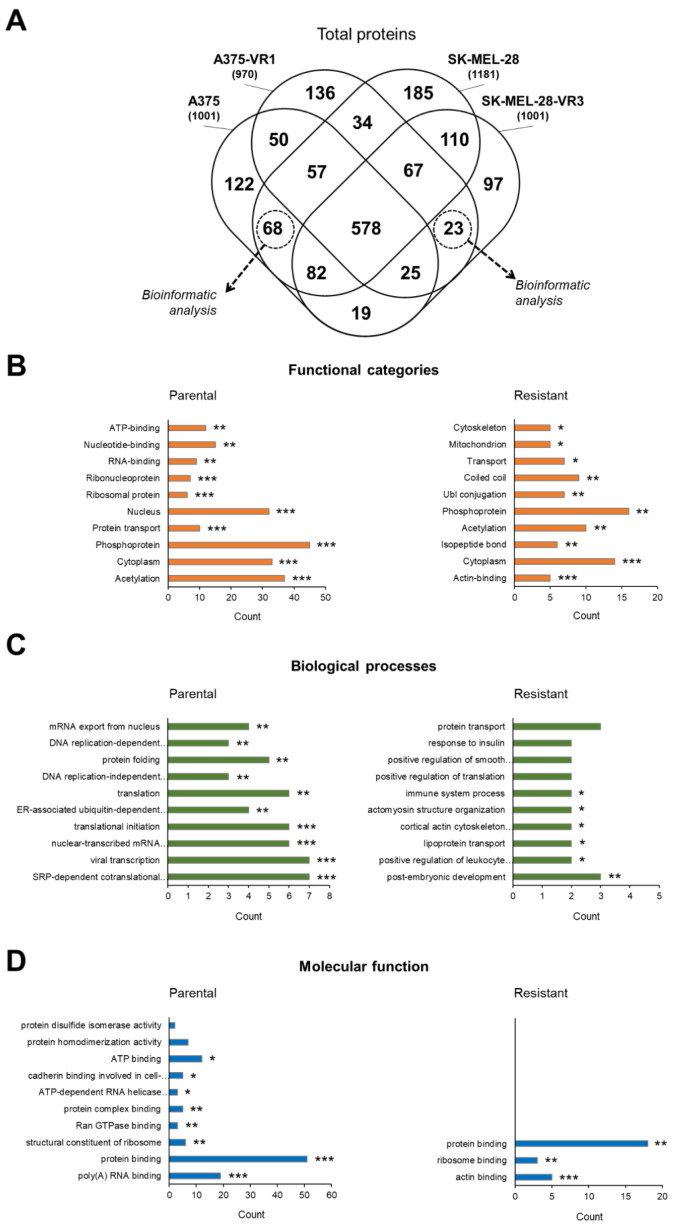
Proteomic analysis of total differentially expressed proteins. (**A**) Venn diagram representing the overlap in the identified proteins in each cell line. A total of 68 and 23 specific common proteins were identified in parental and resistant cells, respectively. Enriched gene ontology (GO) analysis of the identified common specific proteins using DAVID classification based on (**B**) functional categories, (**C**) biological processes, and (**D**) molecular function (count indicates the number of proteins involved in the term). Top 10 terms of DAVID functional annotation chart were shown and ordered according to the statistical significance: * *p*-value < 0.05; ** *p*-value < 0.01; *** *p*-value < 0.001.

To characterize proteins potentially associated with BRAFi resistance, a first level analysis was initially carried out on the total identified proteins, i.e., taking into account the three different denaturation protocols. The mass spectrometry analysis revealed a total of 1001, 970, 1181, and 1001 non-redundant proteins expressed in A375, A375-VR1, SK-MEL-28, and SK-MEL-28-VR3, respectively (Figure 2A). As previously demonstrated, A375 cells show a more aggressive phenotype with higher proliferative rate than SK-MEL-28 cells [29]. To minimize the protein expression difference between A375 and SK-MEL-28 parental cells while highlighting those more likely related to the resistance acquisition, we focused our attention on the specific proteins that were shown to be exclusively expressed only in parental cell lines (68 proteins) or only in resistant sublines (23 proteins) (Table S5 and Figure 2A). These proteins were further analyzed using GO bioinformatics tools. DAVID functional annotation analyses were performed to establish functional categories, biological processes, and molecular function associated with vemurafenib resistance. Analysis according to functional categories revealed few differences between differentially expressed proteins: in parental cells, the specific proteins were associated mostly with ribosomal and RNA-binding proteins, while in resistant cells these were related to actin-binding and post-translational proteins modification (isopeptide bonds and ubiquitination) (Figure 2B). With respect to the characterization according to biological processes, some differences related to DNA/RNA duplication and translational processes, and to molecular transport and immune response for parental and resistant cell lines respectively, were found (Figure 2C). It is interesting to note that differences in the secretion of cytokines/chemokines and in immunomodulation ability in our melanoma resistant model has been recently demonstrated [16]. DAVID classification for molecular function showed only few differences, related to binding features, between parental and resistant cells. This was expected, since our experimental design was specifically focused on highlighting differences due to acquired resistance (i.e., shared behaviors between the two resistant cell lines), and this highly stringent strategy may reduce the number of differences, while strengthening the analysis in terms of revealing the most relevant molecular functions for parental and resistant cells: protein and Poly(A) RNA binding functions for the former and actin binding functions for the latter.

To provide further insights on the protein–protein interaction (PPI) network, the STRING online tool was used. The specific proteins expressed in parental (Figure S2A) and in resistant cells (Figure S2B) were subject to STRING analysis, the results of which highlighted that mammalian target of rapamycin (MTOR) represents a central hub in BRAF-resistant cells, which is in agreement with published studies [30,31]. The presence of MTOR confirmed the efficacy of our model and experimental approach.

### 2.3. Bioinformatic Analysis of DENT1 Differentially Expressed Proteins

**Figure 3:**
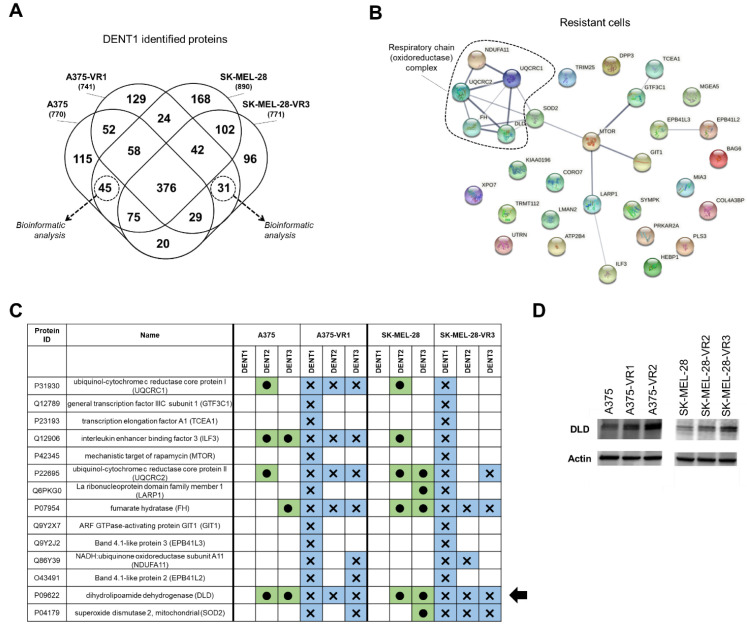
Proteomic analysis of DENT1 differentially expressed proteins. (**A**) Venn diagram representing the overlap in the identified proteins in each cell line. A total of 31 specific common proteins were identified in resistant cells. (**B**) The protein–protein interaction network of vemurafenib-resistant differentially expressed proteins as predicted by the STRING tool (version 11.5; http://string-db.org accessed on 20 July 2022). The links between proteins represent possible interactions (line thickness indicates the strength of association). The relevant pathway of respiratory chain complex was clustered. (**C**) Denaturation pattern of 14 proteins of resistant cells connected in the STRING network. This scheme depicts how these proteins are differently identified by the three denaturation protocols. Black arrow indicates DLD, as the unique protein showing the same identical denaturation pattern in parental (green boxes with circle) and resistant (blue boxes with cross) in both cell lines. (**D**) DLD expression validation by Western blot analysis (actin is used as internal standard).

Since the total proteins analysis revealed many differences between parental and resistant cell lines, most of which were potentially related to cells heterogeneity, a detailed analysis to increase the probability of identifying a more specific resistance-related target was carried out. Different sensitivity to denaturing protocols of protein lysates from resistant and parental cells was analyzed using the TRIDENT approach, which may highlight specific protein-protein interactions, PTMs and/or intra-residues interactions that are otherwise difficult to investigate [26]. We firstly focused our analysis on the identified proteins derived by one specific denaturation protocol (DENT1). As reported in Figure 3A, 45 and 31 specific proteins were expressed only in parental and resistant cells, respectively), both in A375- and SK-MEL-28-derived sublines (see also Table S6). 

GO analysis was performed as previously described, and results were characterized by functional categories (Figure S3A), biological processes (Figure S3B), and molecular functions (Figure S3C). In particular, DAVID analysis with respect to biological processes highlighted the main differences associated with oxidative metabolism in resistant cells (Figure S3B), and these results were corroborated by STRING analysis. In fact, PPI analysis performed on the 31 specific proteins of resistant cells (i.e., peculiar for the resistance phenotype) supported the involvement of mitochondrial oxidoreductase complexes (Figure 3B). Hence, to investigate not only the presence but also the structural/functional behaviors of proteins involved in drug resistance (the aim which the TRIDENT protocol was developed for, see [27] for details), we focused our study on these resistant cells’ specific proteins. A comparative analysis, to assess whether or not the DENT1 proteins had been similarly identified by the other two denaturation protocols (DENT2 and DENT3), was then carried out. Figure 3C shows that the 14 proteins interconnected by the STRING network were differently identified by the three denaturation protocols. It is noteworthy that several of them (namely GTF3C1, TCEA1, MTOR, GIT1, and EPB41L3) were not revealed as resistance-specific by DENT2/DENT3 protocols, while they were identified by DENT1 in both resistant cells but not in either parental one. Furthermore, the structural-functional analysis performed while simultaneously taking into account the DENT1, DENT2 and DENT3 proteomic profiles revealed that DLD showed an identical pattern of denaturation dependence in both A375 and SK-MEL-28 cells and their resistant counterpart (black arrow in Figure 3C), suggesting a key role for its structural-structural behaviors. It is possible that other proteins related to respiratory chain complex grouped in Figure 3B may explain functional differences between BRAFi-resistant and parental cells, but DLD was the only protein showing similar denaturation characteristics shared by both resistant cell types. This trait of the DLD prompted us to investigate it in more detail. 

Therefore, DLD expression in our melanoma cell lines was also evaluated by Western blot analysis in two additional resistant cell lines (namely A375-VR2 and SK-MEL-28-VR2). Figure 3D showed a marked upregulation of DLD in resistant vs. parental cells, both in A375 and SK-MEL-28 cells, suggesting that its enzymatic function could also be important in explaining vemurafenib resistance. Then, we carried out an in silico analysis of DLD peptides identified with mass-spectrometry after TRIDENT protocol application, in order to investigate possible structural differences between parental and resistant cell lines.

### 2.4. Structural Profiling of Dihydrolipoamide Dehydrogenase

**Figure 4:**
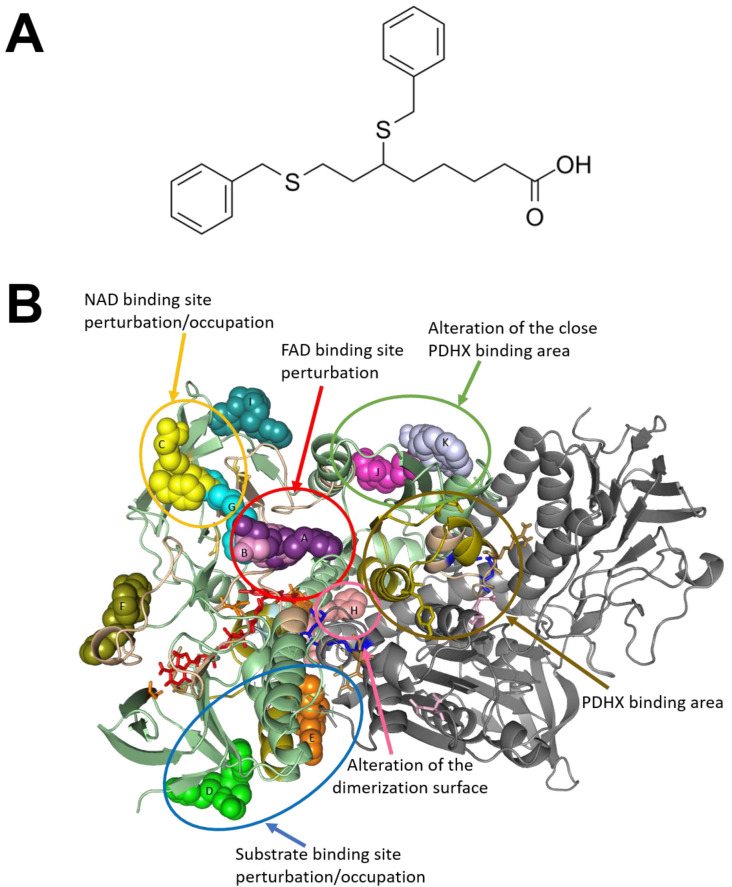
Docking simulations of the interaction of CPI-613 with human DLD. (**A**) The chemical structure of CPI-613, an analogue of α-lipoic acid that inhibits DLD enzyme and targets tumor mitochondrial energy metabolism. (**B**) Pockets detected by docking simulations of the interactions between CPI-613 and human DLD; different circles correspond to different perturbation areas relevant for DLD activity: in spheres, CPI-613 bonded to different sites of human DLD in green (chain A) and grey (chain B). The different positions of CPI-613 are labelled with letters (A to K).

DLD is a flavin adenine dinucleotide (FAD)-dependent enzyme that catalyzes oxidation of dihydrolipoamide into lipoamide with the parallel reduction of NAD+ (nicotinamide adenine dinucleotide) to NADH (FAD is an intermediary in the electron transfer from dihydrolipoamide to NAD+). CPI-613 (6,8-bis(benzylthio)octanoic acid or devimistat) (Figure 4A) is a lipoate analogue able to inhibit DLD activity, and has been investigated in several clinical trials [32]. Docking simulations of the interaction of CPI-613 with human DLD suggested different regions of the protein surface where the small molecules may interact. Figure 4B shows the potential binding regions, labeled with a letter to simplify the discussion of the possible activity of the inhibitor molecule. The binding in two regions may preclude the interaction with FAD, i.e., pocket A and, especially, pocket B; binding at pocket C may occlude the NAD entrance; interaction of the inhibitor with pocket D could induce distortion in the dihydrolipoamide channel, while with pocket E it may occlude the channel for the substrate. Pocket G is the binding site of the NAD. Binding at the pocket H could interfere with the dimerization of the enzyme. Finally, binding to zones J and K could affect the interaction with E3BP or E2b. Details on the functional and cofactor binding sites of DLD are reported under Discussion. 

**Figure 5:**
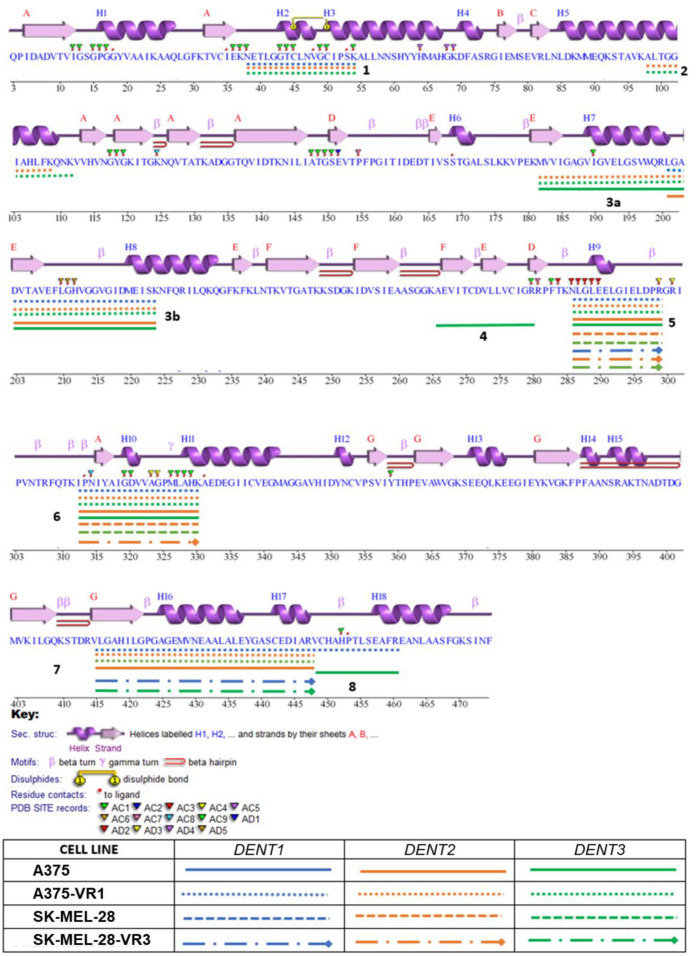
Protein sequence coverage by mass spectrometry of DLD. A visualization of the sequence chain of DLD is presented. Lines correspond to the digested peptides identified (using Proteome Discoverer™ software version 1.4 (Thermo Fisher Scientific, Waltham, MA, USA)) by each of the denaturation treatments of the TRIDENT protocol followed by LC-MS/MS analysis, as previously described [34].

Figure 5 shows the sequence of human DLD with a schematization of the secondary structure and with the position of peptides identified by the proteomic analysis previously described [33]. There are interesting aspects in evidence regarding the position of the identified peptides. Peptide no. 1, in position 38–55, includes the FAD site, and has a partial overlap with the D region identified through the docking simulations. Peptide no.2, in position 98–108, is located in the helix that is part of the channel for dihydrolipoamide. Regions no. 3a and 3b (181–199; 199–224) include the I region identified through the docking simulations. This includes residues important for the binding of NAD. Interestingly, the lysine residues at the extremities are well exposed and accessible for cleavage by protease, in contrast to R199, which is folded towards the inside of the protein structure, making it more difficult to cleave. This may explain the differences in cleavage obtained with the application of different protocols. Peptide no. 4 (266–280) forms part of the NAD binding site, which includes one of the crucial NAD binding residues, G279 (see Discussion for details about amino acids involved in specific functions). Despite the good accessibility of the K265 cleavage site, R280 is shown to be oriented toward the inner of the protein, in a site further back than the rest of the central domain surface, resulting in it being accessible to small molecules such as water, but not to larger molecules such as enzymes, like the trypsin required for the proteomic analysis; this could explain why this peptide is rarely found. Peptide no. 5 (286–300) is an exposed region, and it includes pocket F from the docking simulations. Peptide no. 6 (312–330) is an unfolded region more internal than peptide no. 5, although fluctuation in the long loop may expose the region to protease action. This peptide includes D320, which is part of the FAD binding site. The region of peptide no. 7 (414–447) is at the dimerization interface, and it involves Y438, which interacts with PDHX. The two arginine residues at the cleavage sites are well exposed on the surface, and are therefore easily accessible to the protease. Finally, peptide no. 8 (448–460) is also at the dimerization interface, but is less exposed on the surface. This peptide includes the active site H452.

**Figure 6:**
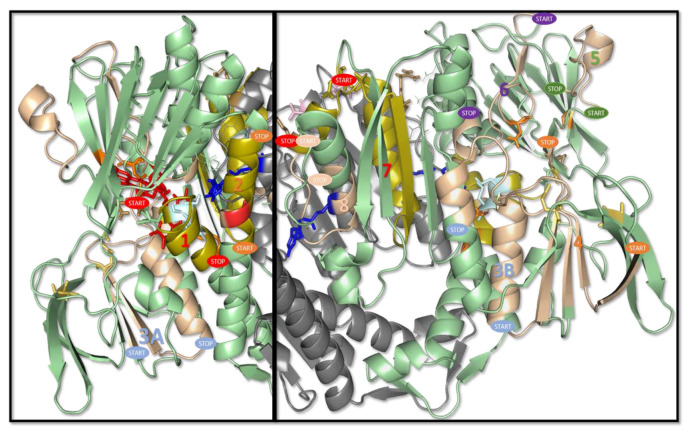
Maps of the detected peptides on the human DLD structure. Each peptide is labelled with its own number and its start and stop position. Peptides 1, 2 and 7 are highlighted in gold.

On the basis of the comparison between peptides found in the parental and resistant cells (Figure 5 and Figure 6), it can be noticed that for A375, the cell lines differ in the identification of peptides 1, 2 (present only in A375-VR1 cells), and 4 (only in the parental A375); for SK-MEL-28, instead, the difference is related to peptide 7, which is present only in resistant cells. Peptides 1 and 2 involve specific sites for which some PTMs are predicted or already known. In particular, upstream and downstream of peptide 1, two glycosylation sites are predicted (N38 and N58), while peptide 2 instead starts and ends with two alternate sites of succinylation/acetylation. 

The major aptitude of DLD of the parental A375 cell line for undergoing post-translational modifications such as succynilation/acetylation/glycosylation, as well as explaining the lack of recognition of these peptides, is also in line with the lower response of this cell to the inhibitor in vitro (see next paragraph). The predicted glycosylation on N38 could interfere with the binding of FAD and with the possible interactions between the inhibitor and the residues in pocket D (responsible for the perturbation of the binding channel). N58 glycosylation, in a similar way, could represent a problem for inhibitor binding in pocket H, as well as for protein dimerization. Even more serious problems arise from the acetylation/succinylation of the peptide 2 terminators, which, being directly involved with the inhibitor binding, can interfere with its interaction in pockets E or D. Peptide 4 is identified only in A375 cells after treatment with DENT3 denaturation. The N-terminal cleavage site Lys265 is well exposed, and is neither annotated as being nor predicted to be a target of acetylation. The C-terminal cleavage site, Arg280, is in a region accessible to the solvent, although it is located in a groove that may not be well exposed to larger molecules. The arginine follows Gly279, a residue involved in the binding of NAD. Moreover, the arginine is followed by a second arginine and a proline, a condition that makes cleavage by trypsin difficult [35]. In fact, the G-R-R sequence has been reported to be a difficult site for trypsin cleavage [35]. Therefore, in the context of the complexity of the factors involved, it is possible that the peptide is recognized only in one cell line and one denaturant condition thanks to the stronger ability under the other conditions to preserve NAD binding and prevent complete exposure to the protease. In the SK-MEL-28 cell lines, in addition to the modifications explaining the absence of these peptides in both lines (parental and resistant), phosphorylation could also be present. In particula3br, the more minor phosphorylation of the DLD produced by the SK-MEL-28-VR3 line compared to the parental one could explain the presence of peptide 7, whose cleavage site (R414) remains unmasked by phosphorylation of the structurally close T412, but could also affect the interaction between DLD and E2B and E3BP, making it weaker. In particular, four sites are predicted to be phosphorylation sites: T205 (in peptide 3b), S441 (in peptide 7), T412 (close to R414) and T545 (peptide 8). 

**Figure 7:**
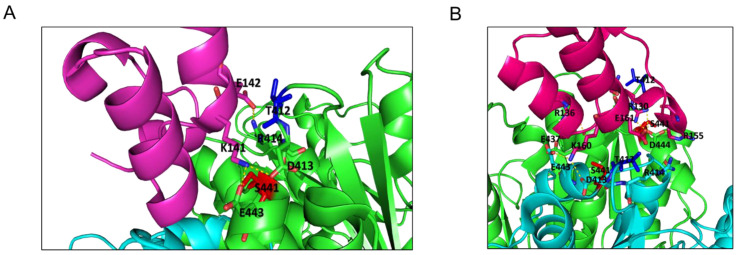
Interactions between human DLD and E2b (panel A) and E3BP (panel B). (**A**) In green ribbon, human DLD; in magenta, E2b (PDB: 3RNM); in red and blue sticks, S441 and T412, respectively; in yellow dots, salt bridges. Phosphate groups on T412 and S441 residues may increase the number of negative charges close to K141, stabilizing the interaction. (**B**) In green and cyan ribbon, human DLD chain A and chain B, respectively; in magenta, E3BP (PDB: 2F5Z); in red and blue sticks, S441 and T412, respectively; in yellow dots, salt bridges between R130 (E3BP) and D444 (DLDH), R136 (E3BP) and E437 (DLDH), R155 (E3BP) and D444 (DLDH), K160 (E3BP) and E443/D413 chain B (DLDH). Phosphate groups on S441 residue on chain A and B may create novel interactions with K160 and R130 respectively; negative charge on T412 may instead modify the orientation of the residue side chains of the loop.

On the basis of the analysis of the interaction between the DLD and rE2b and E3BP (Figure 7A,B, respectively), it is possible to see how an increase in the negative charge by phosphorylation on T412 and S441 may stabilize the interaction with both the molecules. In the E2b-DLD complex (Figure 7A), the formation of three salt bridges is noteworthy, the first between K135 (E2b) and E437 (DLD), the second between E142 (E2b) and R414 (DLD), and the third is possible between K141 (E2b) and D413/ E443 (DLD). T412 and S441 are spatially very close to K141 (distance lower than 4 Å). In the E3BP-DLD complex, on the other hand (Figure 7B), there are four salt bridges: i. R130 (E3BP) with D444 (DLDH_chain A); ii. R136 (E3BP) with E437 (DLDH_chain A); iii. R155 (E3BP) with D444 (DLDH_chain A); and iv. K160 (E3BP) with E4437D413 (DLDH_chain B). The negative charges on S441 on chain A and B may create new interactions with K160 (E3BP) and R130 (E3BP), respectively. Phosphorylation on T412 may instead modify the orientation of the residue side chains of the loop, where it is located with a shift in the R414 side chain, which is becoming closer to T412 and may interact with E161 (E3BP).

### 2.5. Targeting Vemurafenib-Resistant Cells with DLD Inhibitor

**Figure 8:**
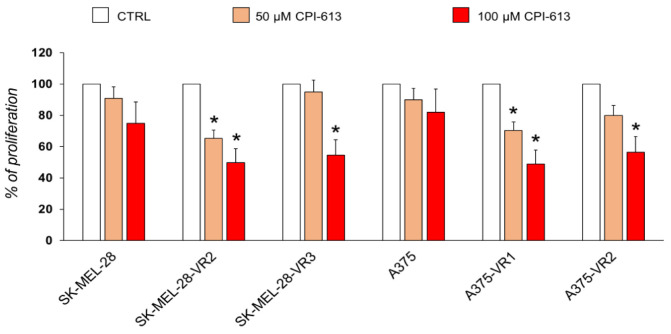
Proliferation rate of melanoma cells untreated (CTRL) or treated with CPI-613 for 48 h. Data are expressed as % proliferation compared to the CTRL (100%) ± SD of three independent experiments (statistical significance: * *p* < 0.05).

Since in silico analysis suggested several differences between resistant and parental cells in terms of DLD structure and its interaction with the inhibitor (CPI-613), these data were integrated with in vitro experiments (also in two additional resistant cells, namely A375-VR2 and SK-MEL-28 VR2 cells). Parental and resistant cells were treated with 50 and 100 µM CPI-613 for 48 h. As shown in Figure 8, the inhibition of DLD significantly reduced the number of cells of all resistant sublines at 100 μM and of three out of four sublines at 50 μM, whereas no significant effect was observed in A375 and SK-MEL-28 parental cell lines.

Cell cycle distribution through flow cytometry was used to investigate the possible mechanism of cell growth inhibition by CPI-613. Exposure to 100 μM CPI-613 resulted in an alteration of cell cycle distribution in melanoma cells (Figure S4). Interestingly, an induction of cell death (sub-G1 population) in A375 resistant cells was observed, with respect to their parental counterpart. Moreover, our data suggest that CPI-613 could suppress the growth of SK-MEL-28 resistant cells through the accumulation of cells in G0/G1, accompanied by a decrease in S and G2/M phases. 

## 3. Discussion

The development of immune checkpoint inhibitors and molecularly targeted drugs has revolutionized the treatment of patients with metastatic melanoma. However, primary resistance and, especially in the case of targeted therapy with tyrosine kinase inhibitors [36], acquired resistance hampers the efficacy of therapy. Although multidrug regimens simultaneously targeting several tumor-related pathways, or the same pathway at different key points, can achieve better results in terms of response rate and survival outcomes, increased toxicity is frequently observed [37]. Moreover, drug resistance occurs in multidrug approaches, too, possibly involving multiple mechanisms with interconnecting effects. Identification of the molecular mechanisms underlying drug resistance is therefore a field of intense investigation that is of paramount importance in the development of new and more effective therapeutic strategies.

In the present study, to identify potential mechanisms of resistance to BRAFi, the proteomic profiles of two extensively studied human melanoma cell lines, namely A375 and SK-MEL-28, and their vemurafenib-resistant sublines were compared. The novelty of our approach lies in taking into account both the expression and the structural-functional behavior of the proteins. This was achieved by applying the TRIDENT protocol, an approach developed primarily to optimize the electrophoretic discrimination of serum proteins related to different sensitivity to denaturation of proteins or protein complexes [27]. In fact, the use of the TRIDENT approach as a pre-prep protocol makes it possible to obtain the following two advantages: (1) to significantly increase the number of expressed proteins by mass spectrometry identification; (2) to highlight many structural and functional features related to the identified proteins. Thanks to this approach, marked differences in electrophoretic profiles, confirmed by the mass spectrometry identification of proteins, were evident within the resistant proteomes compared to the parental ones. This is likely due to specific protein-protein interactions, PTMs, or other tridimensional features, possibly related to the acquisition of drug resistance. The potential key role of PTMs and/or other structural-functional features was demonstrated when each protein cell lysate (from drug resistant or parental cell lines) was denatured under three different protocols, highlighting differences in folding/unfolding balance and their different degrees of accessibility to trypsin degradation, reflected in the significantly different protein expression profiles observed. These striking differences between parental and resistant melanoma cells proteomes were strongly emphasized by this structural approach. Furthermore, our methodology was applied to resistant cell lines (A375-VR1 and SK-MEL-28-VR3) and their own parental counterparts (A375 and SK-MEL-28 melanoma cells) in order to achieve a consensus. This approach turned out to be very useful in minimizing the heterogeneity of melanoma, even regarding BRAFi resistance [38]. It is also important to note that our cellular models possessed different levels of melanin content. In fact, A375 cells are basically amelanotic, whereas SK-MEL-28 cells are lightly pigmented [39], and melanin pigment appears to be an important factor in melanoma growth, metabolism, metastasis, and resistance to radiotherapy [40,41,42]. Furthermore, it has also been reported that MAPKi are associated with the alteration of melanin synthesis [43,44]. Most omics studies focus on the evaluation of gene transcription and/or protein expression, without taking into account the PTMs of expressed proteins, and they are therefore not able to completely evaluate the functionally relevant differences between parental and resistant cancer cells proteomes. To minimize this weakness, our multi-denaturation method [27] was applied, with several modifications, to cell lysates in order to also evaluate the structural-functional behaviors of proteomes expressed by resistant and parental cells.

The bioinformatics analysis revealed that the proteomic patterns of resistant cell lines showed significant differences in terms of molecular function when compared to parental ones, moving from a “proliferative” phenotype (e.g., nucleus, RNA, and nucleic acid binding proteins) towards a more “functional” phenotype (e.g., cytoplasm, actin binding proteins). To exploit the features of the TRIDENT approach, further bioinformatics analyses were carried out, firstly employing the DENT1 protocol. Notably, of the 31 specific proteins identified in resistant cells in both cell lines, only 14 were connected in the STRING analysis (PPI network), and these were therefore selected for subsequent analyses (see Figure 3). When comparing the denaturation patterns derived from DENT1 vs. DENT2/DENT3, several proteins whose structural behaviors were significantly different between the parental and vemurafenib-resistant cells were identified (e.g., ubiquinol-cytochrome c reductase core proteins and interleukin enhancer binding factor 3), but, very interestingly, DLD was the only protein similarly affected by the three denaturation protocols. 

DLD was also found to be markedly over-expressed in all of the resistant sublines under investigation compared to their parental counterparts. It is noteworthy that a pivotal role of this enzyme in melanoma progression was recently suggested by Yumman and colleagues, who demonstrated that DLD down-regulation induced autophagy and inhibited proliferation of melanoma cells [45]. In particular, the human DLD is a homodimeric protein, with a monomer structure of 509 amino acids. The N-terminal segment 1–35 acts as a signal peptide. DLD is organized with an FAD binding domain (36–184), an NAD binding domain (185–317), a central domain (318–385), and an interface domain (386–509). Two cysteine residues, i.e., C45 and C50, constitute the reactive disulfide bridge in proximity to the channel for the access of lipoamide. Other important amino acids for the enzymatic function are H452, which acts as a proton acceptor, and E457, which both act at the channel for the lipoamide of the other monomeric subunit. Three amino acids constitute the FAD binding site, i.e., K54, G119, and D320. Three other amino acids constitute the NAD binding site, i.e., E208, V243 and G279. An important role also attributed to G101, due to its position in the channel for lipoamide, while D413 and Y438 are involved in the interaction with PDHX (pyruvate dehydrogenase complex component X), also known as E3 binding protein, which is responsible for the binding of DLD (E3) to the PDC. Mutagenesis studies have identified reduced activity when K37 and R447 are substituted (this structural and functional information was derived by inspection of the UniProt entry for P09622 and with reference to the 3D structure determination [33]). In the present study, the functional involvement of DLD in melanoma cell resistance to vemurafenib, which has never previously been reported in published studies, is suggested by: (i) the in silico studies, which evidence a different structural accessibility of DLD to trypsin digestion between parental cell lines and drug-resistant sublines; and (ii) the finding that functional inhibition of DLD enzymatic activity induces a significant inhibition of proliferation in all of the resistant sublines under investigation, but not in the parental cell lines. DLD is a key component in several multi-enzymatic complexes, e.g., PDC, which represents a fundamental hub in human metabolism and cellular homeostasis [28]. On the other hand, the metabolic plasticity of melanoma cells is considered a hallmark of disease progression, in addition to being a possible mediator in the acquisition of resistance to targeted therapies [46]. Therefore, the inhibition of DLD activity may induce severe alterations in mitochondrial energetic metabolism. 

CPI-613 (devimistat) is one of the best-characterized DLD inhibitors [33], demonstrating antitumor activity both in vitro and in animal models [47,48]. Furthermore, as a first-in-class mitochondrial metabolism inhibitor, CPI-613 is under clinical investigation for different solid and hematological tumors, both as a single agent and in combination regimens. In this regard, an initial clinical report demonstrated the moderate clinical activity and acceptable toxicity of CPI-613 monotherapy in patients with relapsed or refractory hematological malignancies [49]. Promising activity of CPI-613 in combination with cytarabine and mitoxantrone in patients with refractory/relapsed acute myeloid leukemia was more recently observed in clinical trials [50,51,52]. In phase I clinical trial in patients with newly diagnosed metastatic pancreatic adenocarcinoma, the combination of CPI-613 with a modified FOLFIRINOX regimen was well tolerated and induced an objective response rate of 61% [53]. While the involvement of DLD in melanoma progression has been hypothesized [41], our study demonstrates for the first time that CPI-613, under the specified experimental conditions, impairs proliferation of vemurafenib-resistant clones while only slightly affecting that of the parental cell lines. Interestingly, the effects on cell cycle distribution differ in the two cell lines. Thus, further studies are needed to better understand the mechanism of action of this drug in melanoma cells. CPI-613 was effective on all of the tested resistant sublines (four compared to the parental ones), which we originated starting from two human melanoma cell lines, distinguished not only by possessing different melanin content, as previously stated, but also by their highly different degrees of aggressiveness [29]. These results are consistent with those obtained by structural proteomics and in silico protein modeling analyses. In fact, in addition to the interference in the substrate interaction by binding to pockets D and E, the inhibitor might bind to other functional sites of DLD, such as the NAD site, the FAD site, and through homodimerization and heterodimerization with PDHX interfaces, thus interfering with DLD activity at different levels. These inhibitory effects may be modulated by post-translational modifications. In particular, glycosylation could prevent the inhibitor binding at the substrate site or at the dimerization interface. This could be further evident in parental SK-MEL-28 cells, taking into account the potential phosphorylation highlighted in the Results section. The final evidence could be a gradual inhibitory effect, modulated by the merging of different opportunities of binding with post-translational modifications occurring at different sites in the cell lines. In particular, it is important to underline that the higher efficacy of CPI-613 on resistant sublines may be due to the higher expression of DLD, but also to the different degree of DLD protein folding within the resistant cells, resulting in different degrees of accessibility to the substrate. The bioinformatics analyses carried out on the proteins characterizing the resistant cell lines revealed several other key players and pathways likely functionally involved in resistance phenotype that deserve more in-depth study, and which are presently under further analysis. Moreover, this study is restricted to commercially available melanoma cells; hence, further investigations on patient-derived tissues are needed to strengthen our conclusions.

The combination of TRIDENT and proteomic approaches carried out in the present study show that PTMs and other tridimensional features could provide useful tools and information for the identification of novel potential targets for new therapies in BRAFi-resistant melanomas. In this work, we focused our attention on the DLD, but since it is involved in several important metabolic routes, including mitochondrial α-ketoacid dehydrogenase complexes, in addition to the glycine cleavage system, in-depth studies aimed at better defining which functions would be affected in resistant melanoma cells should be further undertaken.

## 4. Materials and Methods

### 4.1. Chemicals and Reagents

Roswell Park Memorial Institute medium (RPMI-1640), Dulbecco’s Modified Eagle Medium (DMEM), phosphate-buffered saline (PBS), glutamine, penicillin (10,000 UI/mL) and streptomycin (10,000 μg/mL), dimethyl sulfoxide (DMSO), bovine serum albumin (BSA), propidium iodide (PI), and all other reagents were obtained from Sigma Chemicals (St. Louis, MO, USA). Fetal calf serum (FCS) was obtained from HyClone (South Logan, UT, USA). Vemurafenib (PLX4032) was obtained from Selleck Chemicals (Houston, TX, USA), and it was dissolved in DMSO to a stock concentration of 20 mM. 6,8-bis(benzylthio)-octanoic acid (CPI-613), obtained from Tocris Bioscience (Bristol, UK), was dissolved in DMSO (50 mM stock concentration). 

### 4.2. Cell Culture and Generation of Vemurafenib-Resistant Sublines

The BRAF-mutant (V600E) A375 and SK-MEL-28 human melanoma cell lines [54] were obtained from the American Type Culture Collection (ATCC). Moreover, A375 and SK-MEL-28 cells, as reported in our previously published study [29], were selected as the most representative of high and low aggressiveness among 10 different melanoma cell lines. A375 and SK-MEL-28 cells were cultured in DMEM and RPMI-1640, respectively. Cell culture medium was supplemented with 10% FCS, 0.05% L-glutamine, penicillin (100 U/mL), and streptomycin (100 μg/mL), and the cells were maintained at 37 °C in a 5% CO_2_ humidified atmosphere. Vemurafenib-resistant (VR) variants of each cell line (namely A375-VR1, A375-VR2, SK-MEL-28-VR2, and SK-MEL-28-VR3) were derived from original parental cell lines according to a previously published procedure [55] with slight modifications [16]. Resistant sublines were maintained in culture medium containing 2 µM vemurafenib and cultured for one cell cycle in the absence of the drug before each experiment. Cell resistance to vemurafenib was confirmed by using a sensitive and widely accepted cell toxicity assay using the sulphorhodamine B (SRB) assay, as previously described [16,56,57].

### 4.3. Mass Spectrometry Analysis and Protein Identification 

Gel-based proteome analysis was performed on A375, SK-MEL-28, and their first established resistant sublines (A375-VR1 and SK-MEL-28-VR3). Cell lysates were prepared and denatured according to the TRIDENT protocol, as previously described [27]. Briefly, lysates from each cell line were subjected to three different denaturation protocols, namely, DENT1 (dilution 1:1 with PBS), DENT 2 (dilution 1:1 with PBS, boiling at 100 °C for 2.5 min), and DENT3 (dilution 1:1 with 18% *w*/*v* mannitol solution, sonication for 60 min then followed by dilution 1:1 with sample buffer (SB) without bromophenol blue, and boiling at 100 °C for 2.5 min). The last two protocols were selected for their stronger denaturation activity. All three pre-treated samples were mixed in 1:1 ratio with SB (44 mM tris-HCl pH 6.8, 2% SDS (*w*/*v*), 10% glycerol (*v*/*v*), 5% 2-bmercaptoethanol (*v*/*v*) and 0.0125% bromophenol blue (*w*/*v*)), and then heated for 7 min in a thermoblock pre-heated at 95 °C (Thermomixer Compact by Eppendorf, Hamburg, DE). The product of each denaturation protocol was then subjected to SDS-PAGE. The same amount of total protein extracts (20 µg for DENT1, DENT2, and DENT3 pre-treated samples) were loaded onto a 4–15% Mini-PROTEAN^®^ TGX™ pre-cast polyacrylamide gel (Bio-Rad Laboratories, Hercules, CA, USA), and the electrophoretic profile was evaluated using a colloidal blue staining kit (Invitrogen; Thermo Fisher Scientific, Inc., Waltham, MA, USA) according to the manufacturer’s instructions. Then, each protein band was cut, and the proteins were reduced (dithiothreitol), alkylated (iodoacetamide), and digested overnight with bovine trypsin sequencing grade (Roche Applied Science, Monza, Italy) as described elsewhere [27]. The peptide mixtures were analyzed by nano-reversed-phase liquid chromatography-tandem mass spectrometry (RP-LC-MS/MS), and proteins were identified using Proteome Discoverer software (version 1.4, Thermo Fisher) as previously described [34]. 

### 4.4. Bioinformatics Analyses

The DAVID (Database for Annotation, Visualization and Integrated Discovery; version 6.8) online tool (http://david.abcc.ncifcrf.gov/) (accessed on 15 December 2021) [58] was used to characterize the differentially expressed proteins according to the gene ontology (GO) terms molecular function (GOTERM_MF_DIRECT), biological processes (GOTERM_BP_DIRECT), and functional categories (UP_KEYWORDS) [59]. The Search Tool for the Retrieval of Interacting Genes (STRING; version 11.5) (http://string-db.org) (accessed on 20 February 2022) [60] was used to evaluate functional protein-protein interactions. The interaction networks were obtained on the basis of confidence scores (threshold score 0.4). 

### 4.5. Immunoblotting

Whole cell lysates were separated on 4–15% Mini-PROTEAN^®^ TGX™ pre-cast polyacrylamide gel (Bio-Rad) and blotted onto Nitrocellulose using Trans-Blot^®^ Turbo™ Transfer Starter System (Bio-Rad). After blocking for 1 h in 10% BSA in tris-buffered saline Tween buffer, membranes were probed with antibodies specific for DLD (Sigma-Aldrich) and actin (Novus Biologicals, Littleton, CO, USA). Horseradish peroxidase-conjugated goat anti-rabbit IgG and goat anti-mouse secondary antibodies (Santa Cruz Biotechnology, Santa Cruz, CA, USA) were used, followed by visualization with a Clarity Western ECL Substrate Kit (Bio-Rad). Chemiluminescence was revealed by means of a FluorChem System (Cell Biosciences, Santa Clara, CA, USA), and digitized images were used for purposes of densitometric quantification.

### 4.6. In Silico 3D Structure Analyses

The protein structures were obtained from the Protein Data Bank (PDB) [61]. Different PDB files were used for the analyses, depending on which was the most suitable for the application. Docking simulations were performed using the PDB structure 6I4Q [33], which was preferred to the others because it is more complete, binds FAD coenzyme, and has the highest resolution (R = 1.75). Docking simulations were performed between the protein and the ligand 6,8-bis(benzylthio)octanoic acid (CPI-613), the structure of which was downloaded from the PubChem database [62] using AutoDock 4.2 [63]. The PDB files with the codes 3RNM [64] and 2F5Z [65] were used to investigate the complex structures of DLDH with E2b (subunit-binding domain of the branched-chain alpha-ketoacid dehydrogenase complex) and E3 binding protein, respectively. The PDBePISA tool (Proteins, Interfaces, Structures and Assemblies; https://www.ebi.ac.uk/pdbe/pisa/) (accessed on 15 July 2022) was used to detect the interaction at the protein-protein interface. Visualization of the 3D structures was performed using the open source PyMOL Molecular Graphics System (https://sourceforge.net/projects/pymol/) (accessed on 15 July 2022), Version 2.0 Schrödinger, LLC. A visualization of the sequence chain with a schematization of the secondary structure, functional amino acids, and the identified fragments based on the PDBsum diagram (http://www.ebi.ac.uk/pdbsum/) (accessed on 15 July 2022) was produced. Other information was retrieved from the UniProt database (http://www.uniprot.org) (accessed on 15 July 2022) and using the ScanProsite tool (https://prosite.expasy.org/scanprosite/) (accessed on 15 July 2022).

### 4.7. Cell Proliferation and Cell Cycle Analysis

To perform the proliferation studies, melanoma cells were seeded onto 12-well plates (4 × 10^4^ cells per well) and treated with CPI-613 (50 and 100 µM) for 48 h or vehicle as a control, and then harvested and counted using a Neubauer-modified chamber. For cell cycle distribution analysis, cells were exposed to 100 µM CPI-613 for 24 h. Cells were harvested and fixed in 80% cold ethanol, then washed and incubated with 200 µg/mL ribonuclease A (Life Technologies) for 30 min at 37 °C and 50 µg/mL PI. Samples were analyzed with a FACSCanto Becton Dickinson Instrument (Becton Dickinson, San Jose, CA, USA) and FACSDiva software (5.0.3 version).

### 4.8. Statistical Analysis

The results are expressed as the means of three independent experiments ± standard deviations (SD). The statistical significance of the differences was determined by two-tailed *t*-tests; the significance threshold was set at *p* ≤ 0.05.

## 5. Conclusions

The metabolic plasticity of melanoma cells is considered to be of paramount importance in explaining the acquisition of resistance to antineoplastic therapies. The use of energy sources alternative to glucose (i.e., amino acids) is linked to high mitochondrial oxidative metabolism in targeted-therapy-resistant melanoma [66]. Therefore, our proteomic, structural, and functional results strongly suggest that DLD, as a key enzyme in several multi-enzymatic complexes involved in mitochondrial energy metabolism, may represent a novel and suitable target for the treatment of BRAFi-resistant melanoma. Although our in vitro experimental approach limits the generalizability of our conclusions to in vitro studies only, it is noteworthy that devimistat is presently under intense investigation in Phase II and III clinical studies (https://clinicaltrials.gov/) (accessed on 28 October 2022), supporting its low in vivo toxicity.

## Data Availability

Data available on request by contacting the corresponding author.

## Supplementary Materials

The following supporting information can be downloaded at: https://www.mdpi.com/article/10.3390/molecules27227800/s1, Figure S1: Proliferation curve of A375 parental and resistant (VR1 and VR2) cells in the presence of vemurafenib; Figure S2: The protein-protein interaction network of differentially expressed proteins as predicted by the STRING software. The links between proteins represent possible interactions (line thickness indicates the strength of association). The two significant pathways for parental cells were clustered; Figure S3: Enriched gene ontology (GO) analysis to the DENT1 identified common specific proteins using DAVID classification based on (A) functional categories, (B) biological processes, and (C) molecular function (count indicates the number of proteins involved in the term). Top 10 terms of DAVID functional annotation chart were shown and ordered according to the statistical significance: * *p*-value < 0.05; ** *p*-value < 0.01; *** *p*-value < 0.001; Figure S4: Flow cytometric analysis of floating and adherent cells. Graph shows cell cycle of melanoma cells treated or not with CPI-613; Table S1: List of proteins identified by proteomics analysis in A375 melanoma cells; Table S2: List of proteins identified by proteomics analysis in A375-VR1 melanoma cells; Table S3: List of proteins identified by proteomics analysis in SK-MEL-28 melanoma cells; Table S4: List of proteins identified by proteomics analysis in SK-MEL-28-VR3 melanoma cells; Table S5: Specific proteins commonly expressed in parental and resistant cells; Table S6: Specific DENT1 proteins commonly expressed in parental and resistant cells.

## Sample Availability

Samples of the compounds are not available from the authors.
