# Supplementary material for: Identification of Dihydrolipoamide Dehydrogenase as Potential Target of Vemurafenib-Resistant Melanoma Cells
Source: Molecules. 2022 Nov 12;27(22):7800. doi: 10.3390/molecules27227800 (PMC9698468; doi:10.3390/molecules27227800)
Supplement: Supplementary file 1 [file molecules-27-07800-s001.zip › Supplementary Material (Figures) - Revised.pdf]

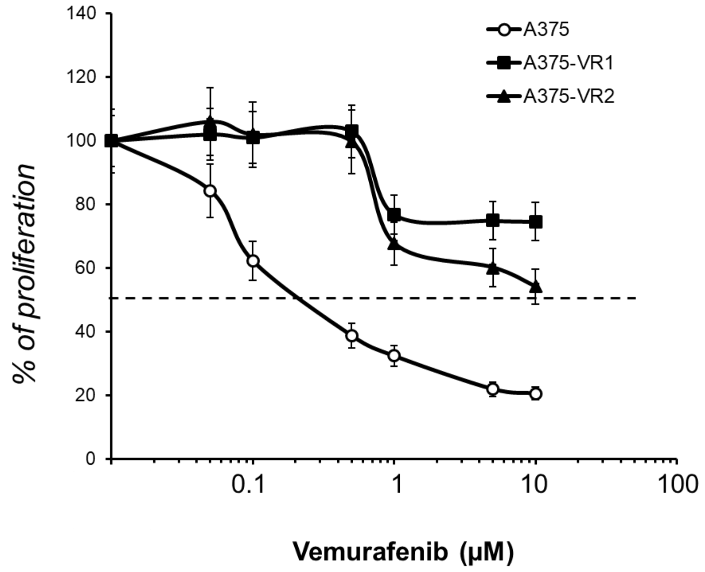

Figure S1. Proliferation curve of A375 parental and resistant (VR1 and VR2) cells in the presence of vemurafenib.

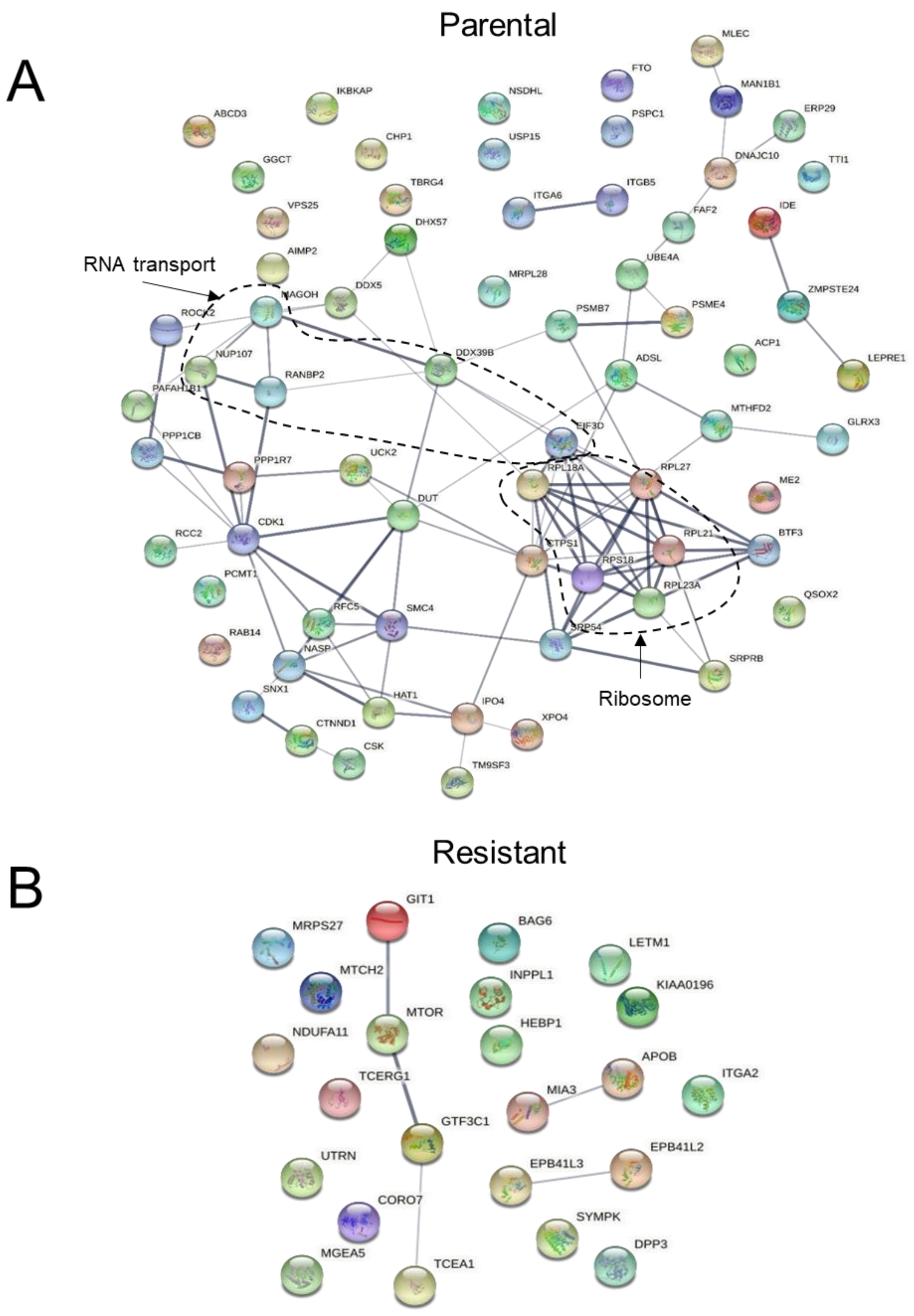

Figure S2. The protein–protein interaction network of differentially expressed proteins as predicted by the STRING software. The links between proteins represent possible interactions (line thickness indicates the strength of association). The two significant pathways for parental cells were clustered.

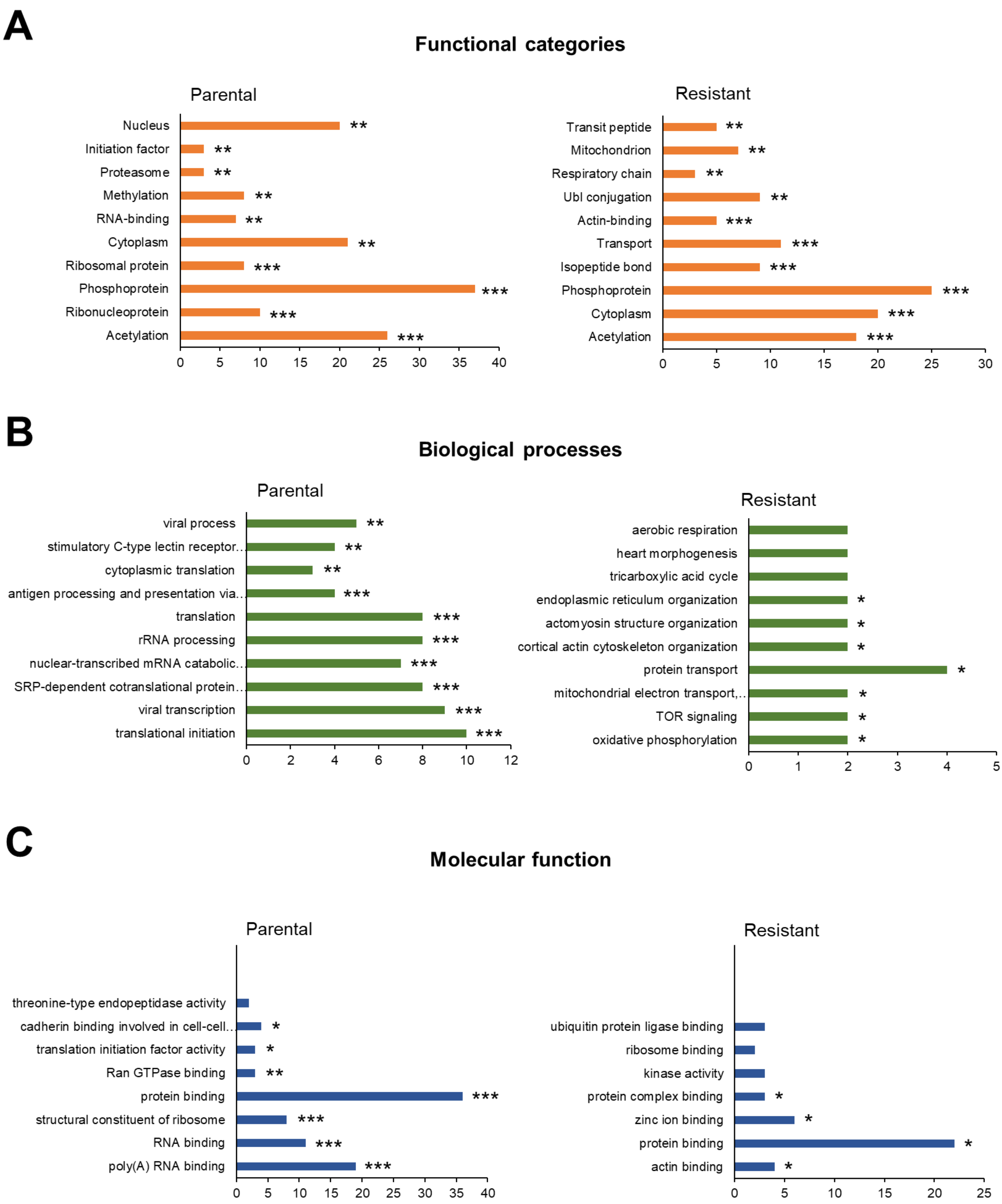

Figure S3. Enriched gene ontology (GO) analysis to the DENT1 identified common specific proteins using DAVID classification based on (A) functional categories, (B) biological processes, and (C) molecular function (count indicates the number of proteins involved in the term). Top 10 terms of DAVID functional annotation chart were shown and ordered according to the statistical significance: * P-value < 0.05; ** P-value < 0.01; *** P-value < 0.001.

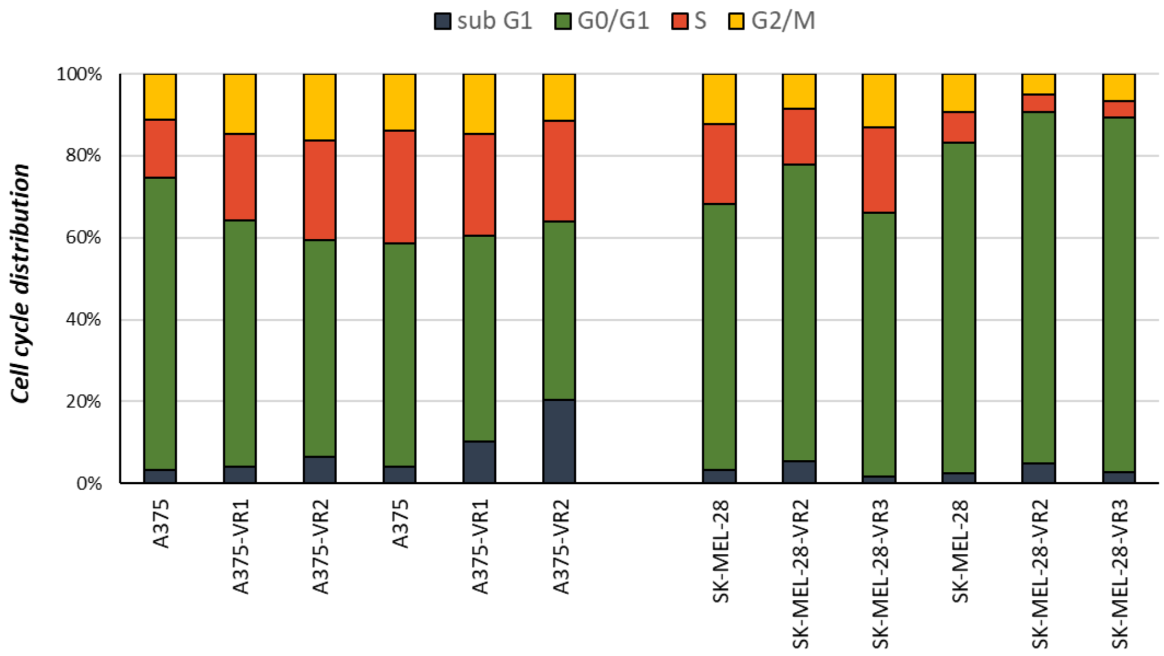

CPI-613	-	-	-	+	+	+	-	-	-	+	+	+

Figure S4. Flow cytometric analysis of floating and adherent cells. Graph shows cell-cycle distribution of melanoma cells treated or not with CPI-613.
